# Evidence-based case report: How to deal with unpredicted endometriosis nodule closed to ureter and rectum during laparoscopy

**DOI:** 10.1016/j.ijscr.2019.07.012

**Published:** 2019-07-22

**Authors:** Sigit Purbadi, Bella Aprilia, Lisa Novianti

**Affiliations:** a Division of Oncology, Department of Obstetrics and Gynecology, Faculty of Medicine University of Indonesia/Dr. Cipto Mangunkusumo Hospital, Salemba Raya Street No.5, Kenari, Senen, 10430, Jakarta, Indonesia; b Department of Obstetrics and Gynecology, Faculty of Medicine University of Indonesia/Dr. Cipto Mangunkusumo Hospital, Salemba Raya Street No.5, Kenari, Senen, 10430, Jakarta, Indonesia; c Faculty of Medicine Gadjah Mada University, Farmako Street, Depok, Sleman, Jogjakarta 55281, Indonesia

**Keywords:** Rectovaginal endometriosis, Surgical ablation, Surgical ablation, Laparoscopy

## Abstract

**Introduction:**

Endometriosis is characterized by the growth of endometrial-like tissue within and outside the pelvic cavity. Peritoneum nodules invaded more than 5 mm representing the commonest form of deep infiltrating endometriosis nodules might challenge inexperienced operator due to its location near ureter and the rectum. The aim is to provide important steps on how to deal with unexpected peritoneal endometrial nodules located closed to ureter and rectum.

**Presentation of case:**

A 43-year-old female underwent laparoscopic cystectomy after being diagnosed to have right endometriosis cyst. The researchers found multiple endometriosis nodules located closed to rectum and ureter after performing cystectomy. A search was conducted on PubMed® with the keywords of “Peritoneal endometriosis nodule” AND “rectovaginal endometriosis nodule” AND “Surgical ablation” OR “Surgical excision” AND “Laparoscopy” AND “Pelvic pain”. Reference lists of relevant articles were searched for other possible relevant studies. After selecting the articles, the critical review was performed based on a standardized appraisal form for the treatment study.

**Discussion:**

Three eligible studies were appraised to assess the surgery outcome (dyspareunia), based on ablation and excision criteria. The pain was decreased during 6 months of follow up, with no difference in both techniques. The minimal requirement to remove the posterior nodules is knowledge of pelvic retroperitoneal anatomy.

**Conclusion:**

In all endometriosis cases which require surgery will need to be performed by an experienced operator. If rectovaginal endometriosis nodule was unexpectedly found during intraoperative and recognition of rectum and ureter must be done, knowledge of retroperitoneal anatomy is required.

## Highlights

• Endometriosis surgery decreased dyspareunia during 6 months follow up.
• No difference outcome between excision surgery and ablation surgery.
• Removal posterior nodules should be performed by experienced operators.
• The minimal requirement is knowledge about retroperitoneal anatomy.

## 1 Introduction

Endometriosis is a chronic gynecological disorder that is characterized by the growth of endometrial-like tissue within and outside the pelvic cavity, primarily in the ovaries and pelvic peritoneum. Ovarian lesions are characterized by cysts with hemorrhagic content. While endometriosis nodules can manifest as invasive tissue that infiltrates structure more than 5 mm from the peritoneal surface deep infiltrating endometriosis (DIE) and mostly cause chronic pelvic pain. It was found that 20–50% reproductive women with infertility, 90% with chronic pelvic pain, and 25% asymptomatic women were accidentally discovered either during laparoscopy or laparotomy [1], [2]. A definitive diagnosis of endometriosis is based on histology confirmation of surgically resected lesion containing endometrial glands and stroma with various amounts of inflammation and fibrosis.

Posterior nodules represent the commonest form of DIE nodules which require real operative challenge due to common involvement of vital retroperitoneal structures (ureter, bowel, vessels, and nerves). Laparoscopy appears to be the ideal tool to perform such surgery, offering the advantages of magnification, accurate hemostasis, precise dissection, and careful handling of delicate tissue. Nevertheless, laparoscopic management of retroperitoneal endometriosis should not be undertaken by inexperienced operators and thorough knowledge of pelvic retroperitoneal anatomy is a prerequisite for radical and uncomplicated removal of DIE nodules [4]. Here, the research aim is to provide important steps on how to deal with unexpected peritoneal endometrial nodules located in rectovaginal space near the rectum. The work has been reported according to the SCARE criteria [11].

## 2 Presentation of case

**Fig. 1:**
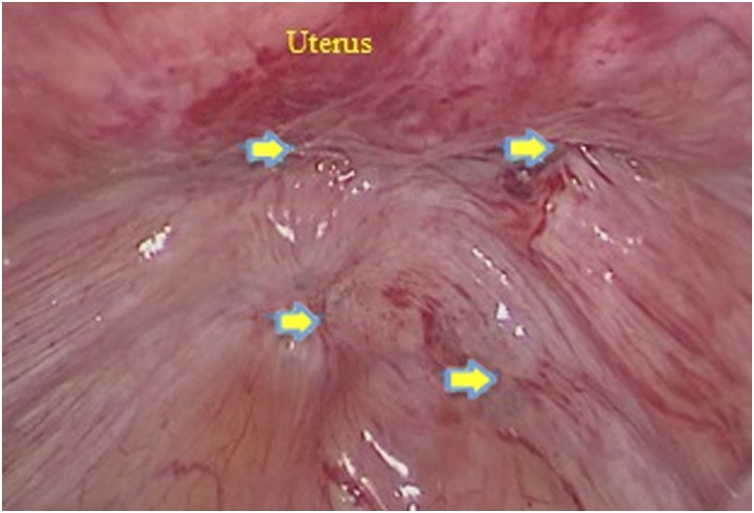
Multiple endometriosis nodule (yellow arrows) sized 1 to 1.5 cm were found in rectovaginal space closed to rectum and ureter.

**Fig. 2:**
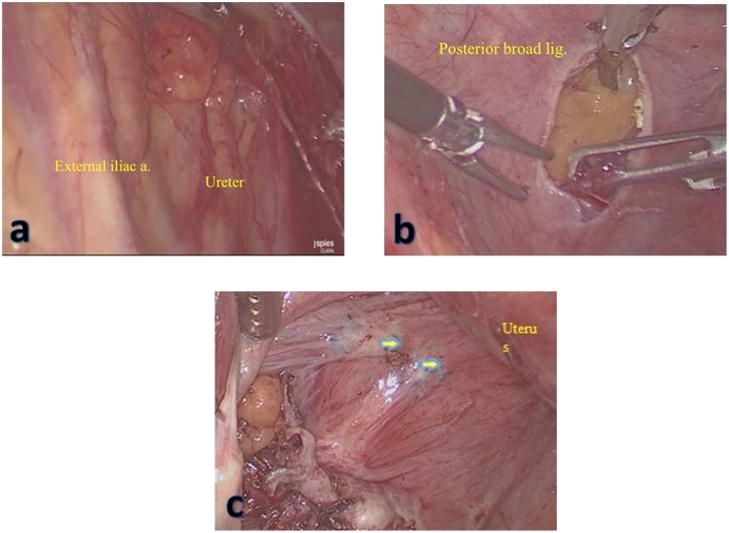
Identifying the ureter via transparam peritoneum layer (a). Opening posterior broad ligament trough retroperitoneal space (b). After identify the ureter, all of endometriosis should be excised (c).

**Fig. 3:**
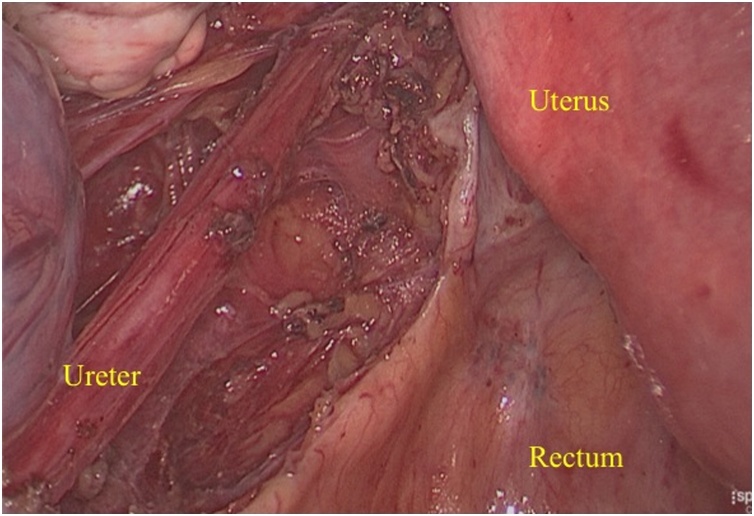
Final view after ureter dissection and endometriosis nodule excision.

**Fig. 4:**
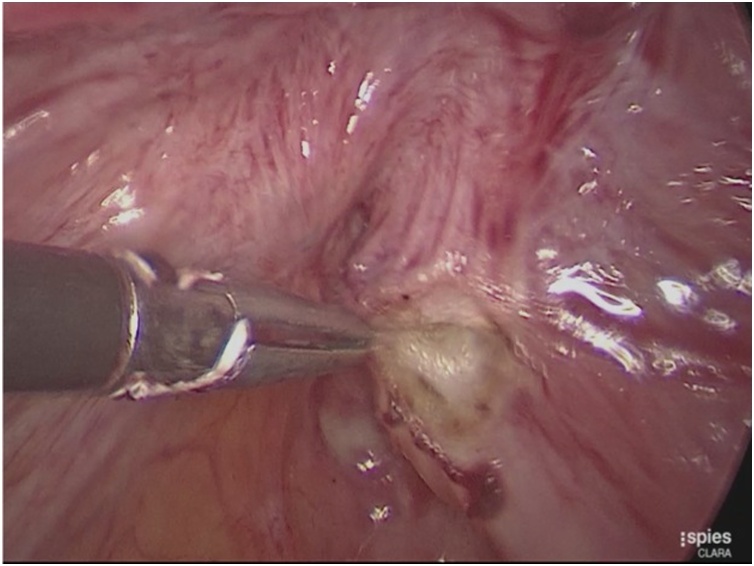
Peritoneal surface of the rectum was excised, fat layer was still intact, countinue with endometriosis nodule excision.

A 43−years − old female patient came with chief complaint abdominal mass for 1 year before admission. On the physical examination and ultrasound examination, she was diagnosed with right endometriosis cyst sized 10 × 12 × 10 cm. She underwent laparoscopy cystectomy after preoperative preparation [12]. Pneumoperitoneum was achieved using a 10 mm port direct trocar insertion until an intra-abdominal pressure of 15 mmHg was reached. The researchers first placed a midline supraumbilical 10 mm port for the telescope, then two 5 mm accessory trocars were positioned in the left and right lateral quadrants visualized via a 10 mm telescope inserted through the supraumbilical port. The left and right accessory ports and trocars were inserted laterally to her deep inferior epigastric arteries. Intraoperative findings, uterus, both tubes, and left ovary were within normal. Right ovary was enlarged, adhered to lateral uterine corpus when the researchers performed adhesiolysis using harmonic ultrasonic scalpel (Ethicon Endo-Surgery Inc,). Then, chocolate fluid came out, corresponding to endometriosis cyst. After performing cystectomy, the researchers found multiple endometriosis nodules sized 1 to 1.5 cm, located in rectovaginal space close to rectum and ureter (Fig. 1). First, the researchers opened the peritoneum surface of the rectum and found the intact fat layer. Therefore, the researchers excised the endometriosis nodule easily (Fig. 2). The procedure was continued by opening posterior broad ligament to identify the ureter (Fig. 3). After the researchers dissected the ureter from posterior broad ligament, they excised the remaining endometriosis nodules safely (Fig. 4). Harmonic and bipolar forceps were used on most of the procedure. Histopathology exam has confirmed the presence of hemosiderin-laden macrophages and endometrial glands [[13], [14], [15]].

## 3 Clinical questions

What is the best decision in unexpected peritoneal endometriosis nodule located in rectovaginal space undergoing laparoscopy?

## 4 Search strategy

**Fig. 5:**
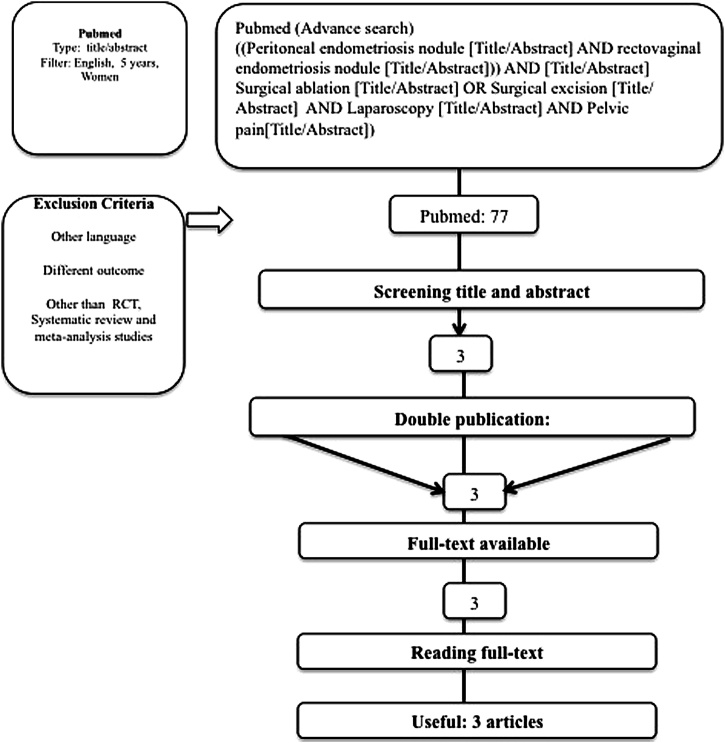
Searching flow.

The search was conducted on December 8^th^ 2018 on the PubMed® with the keywords of “Peritoneal endometriosis nodule” AND “rectovaginal endometriosis nodule” AND “Surgical ablation” OR “Surgical excision” AND “Laparoscopy” AND “Pelvic pain” with certain techniques (Fig. 5). The search focused on articles in surgical technique showing the outcome of the studies. After obtaining a result, the first selection was done by screening the study titles and abstracts. Three articles were available as full text (Table 1), and all of them were included in the analysis.

**Table 1:** Three eligible studies.

Reference	Design	Required parameters	Result
Healey M, et al [5]	RCT, double-blinded	VAS questionnaire before and after surgery Follow up every 3 months for 1 year and 6 months every 5 year	Dyspareunia decreased 12 months compared to before surgery and 60 months compared with 12 months on both groups (p 0.03 and p 0.007)
Pundir J, et al [6]	Systematic Review and Meta-analysis	3 RCT studies were eligible Follow up at 3, 6, and 12 months	Dyspareunia RR 0.96 [0.07–1.99]
Riley K et al [7]	RCT	VAS scoring at 6 and 12 months Secondary outcomes: 6 and 12 months from SF-12 score, PISQ-12 score, Intnat’l Pelvic Pain score	Dyspareunia at 6 months (MC, −22.96; 95% CI −39.06 to −6.86; p = 0.01)

VAS: Visual Analogue Scale; SF-12: Short Form Health Survey; PISQ-12: POP/Urinary Incontinence Sexual Function Questionnaire; Intnat’l Pelvic Pain Score: International Pelvic Pain Assessment.

## 5 Critical appraisal

Appraisal of 3 eligible studies used critical appraisal questions developed by the Centre of Evidence-Based Medicine (CEBM), University of Oxford (available at http://www.cebm.net/critical-appraisal/).

## 6 Discussion

A definitive diagnosis of endometriosis can be made with laparoscopic evaluation. Patients may have symptoms such as dyspareunia or dyschezia which is not classic endometriosis type symptom that prevents from receiving the diagnosis and subsequent treatment. Surgical management has a role in the treatment of endometriosis. It was believed that excision surgery will improve pain outcomes [3]. However, studies showed no difference outcome between ablation and excision [[5], [6], [7]].

Laparoscopic management of retroperitoneal endometriosis should be performed by experienced operators. When facing endometriosis nodule which is the closed to are rectum and ureter, we must excise the nodule without any injury on the ureter and rectum. The surgical technique must start to achieve the retroperitoneal, release the ureter from peritoneum and finally you can excise the nodule. The dissection should continue to identify the nodule whichis cloded to the rectum. Identify the nodule, fat layer and the rectum. If fat layer metabolized we must identify between nodule and the rectum. There are layers on rectum that should be noticed during surgical excision [8].

• The anterior superior part of rectum covered with peritoneum and rest is extraperitoneal in contact with endopelvic fascia. In this case, the researchers can identify the intact fat layer. It was high likely endometriosis nodule located superficially.
• Muscularis: outer longitudinal layer (3 bands of colic taeniae coli merge into a continuous layer at the rectosigmoid junction, down through sphincter level), inner circular muscle, Auerbach’s plexus
• Submucosa: Meissner’s plexus (neuronal network) and strongest layer (connective tissue)
• Mucosa: muscularis mucosa, lamina propia, epithelium

**Fig. 6:**
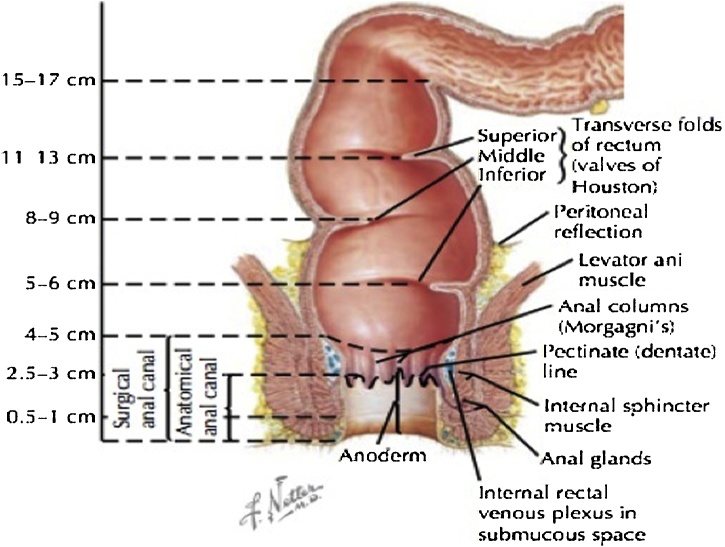
Anorectal anatomy^8^.

Due to the anatomical challenge, minimal requirement during the laparoscopic learning curve is knowledge of pelvic retroperitoneal anatomy (Fig. 6). If inexperienced operators unexpectedly found endometriosis nodule during laparoscopy, the thing should be considered is whether the operator has knowledge about retroperitoneal anatomy. Thus, they can decide whether the surgery should resect some organ or dissect the nodule from an organ without injury.

There are two methods in surgically treating peritoneal endometriosis. It is done by surgical excision or ablation. Surgical procedures are usually carried out by some form of energy such as monopolar or bipolar electrocauterization. Some techniques involve laser technology use, such as YAG, KTP or CO_2_ laser to vaporize or excise endometriosis lesions. Whereas, some other approaches are simply based on a cold scissor technique to excise lesions [9]. Ablation is primarily performed using diathermy. It has been associated with increased risk of thermal injury to surrounding tissue and is often incomplete because of the limited depth of penetration in DIE [5].

In this case, the researchers performed surgical excision after identifying the rectum and ureter. There are two possibilities surgical approach to identify ureter; by opening retroperitoneal space from anterior broad ligament or dissecting the ureter from posterior broad ligament if the ureter is severely adhered (which is what we did in this operation). After ureter was dissected, they can easily remove the endometriosis nodule safely. Then they carefully excised the peritoneum surface of the rectum. After they found that the fat layer was intact, they ensured that this peritoneal endometriosis can be removed safely. However, if the nodule had infiltrated the fat layer, they would have to find the longitudinal muscular as the outer layer of the rectum.

When speaking about DIE, it means more than one tissue has been infiltrated (peritoneum and deeper retroperitoneal structures such as muscular or mucosa of the bowel, fatty tissue of retroperitoneum, umbilicus, nerves, ureter, bladder, sacrouterine ligament, rectovaginal space, and vagina, diaphragm or pericardium) [[16], [17], [18]]. The decision-making to the approach and modality of surgical treatment is subject to the location of endometriotic lesions since posterior location nodules must be resected by carefully pelvic plexus nerve –sparring, bowel, and ureter resection [10].

## 7 Conclusion

All women with endometriosis and who require surgery need an experienced operator. If rectovaginal endometriosis nodule is found during intraoperatively, the recognition of rectum and ureter must be done. Thus, knowledge of retroperitoneal anatomy is required. Endometriosis nodule can be performed with both surgical ablation and excision, but excision is considered as the safest way because mostly ureters adhere to posterior part. A preoperative examination using a physical exam is very important, combined with imaging from transvaginal and transrectal ultrasound examination and MRI.

## Funding

This research did not receive any specific grant from funding agencies in the public, commercial, or not- or-profit sectors

## Ethical approval

Ethical or review board approval was not required for this case report in our institution, Dr. Cipto Mangunkusumo Hospital, Jakarta, Indonesia.

## Consent

Written informed consent has been obtained from the patient for the publication of this case report and any accompanying images.

## Author contribution

The laparoscopy cystectomy was performed by Dr. Sigit. The case report was created by Dr. Bela. The final editing and proofreading was done by Dr. lisa, Dr. Bela, and Dr. Sigit.

## Registration of research studies

None. This was not a research study.

## Guarantor

The guarantor for this case report is Dr Sigit Purbadi.

## Provenance and peer review

Not commissioned, externally peer-reviewed.

## Declaration of Competing Interest

The authors have no conflict of interest to declare.
